# The presence of erosive joints is a strong predictor of radiological progression in hand osteoarthritis: results of a 2-year prospective follow-up of the Liège Hand Osteoarthritis Cohort (LIHOC)

**DOI:** 10.1186/s13075-020-02390-x

**Published:** 2021-01-06

**Authors:** Audrey Neuprez, Jean-François Kaux, Médéa Locquet, Charlotte Beaudart, Jean-Yves Reginster

**Affiliations:** 1 Division of Public Health, Epidemiology and Health Economics University of Liège, WHO Collaborating Centre for Public Health Aspects of Musculoskeletal Health and Agin, Liège, Belgium; 2 Rehabilitation and Sports Traumatology Department, University and University Hospital of Liège, Liège, Belgium; 3 Chair for Biomarkers of Chronic Diseases, Biochemistry Department, College of Science, King Saud University, Riyadh, Kingdom of Saudi Arabia

**Keywords:** Disease progression, Erosive, Hand, Osteoarthritis

## Abstract

**Background:**

This study measured the magnitude and determinants of clinical and radiological progression in patients with hand osteoarthritis (HOA) over a 2-year prospective follow-up to gain a greater understanding of the disease time course.

**Methods:**

Two hundred three consecutive outpatients diagnosed with HOA were followed for 2 years (183 women, median age 69 years). Pain and function were evaluated using the Australian/Canadian Osteoarthritis Hand Index (AUSCAN), and clinical examination recorded the number of painful/swollen joints and nodes. X-rays were scored using Kellgren-Lawrence (KL) and Verbruggen-Veys scales. Clinical progression was defined as deterioration in AUSCAN ≥ the minimal clinically important difference. Radiographic progression was defined as (a) one new erosive/remodeled joint, (b) progression of ≥ one anatomical stage in one joint, or (c) change in KL total score above the smallest detectable difference. Logistic regression was performed to determine whether patient characteristics influenced clinical and radiological progression.

**Results:**

After 2 years, all radiographic scores deteriorated significantly in the study population (*p* <  0.05), and the number of proximal and distal interphalangeal nodes was significantly higher (*p* <  0.01). The AUSCAN, number of painful joints at rest or at pressure, number of swollen joints, and pain measure on a visual analog scale remained unchanged. At the individual level, the number of patients with clinically meaningful progression ranged from 25 to 42% (clinical progression) and from 22 to 76% (radiological progression). The only significant predictor of worsening of total AUSCAN was AUSCAN pain subscale < 74.5 (odds ratio [OR] 1.02 [1.01, 1.03]; *p* <  0.01). The presence of ≥ four swollen joints (OR 2.78 [1.21, 6.39]; *p* = 0.02) and erosive osteoarthritis (OR 13.23 [5.07, 34.56]; *p* <  0.01) at baseline predicted a new erosive joint. A meaningful change in KL was more frequent with painful joints at baseline (OR 3.43 [1.68, 7.01]; *p* <  0.01).

**Conclusions:**

Evidence of radiological progression over 2 years was observed in patients with HOA in the LIHOC population even without clinical worsening of disease. For individual patients, baseline pain level is predictive for clinical progression and the presence of erosive or swollen joints are significant predictors of radiological progression.

**Supplementary Information:**

The online version contains supplementary material available at 10.1186/s13075-020-02390-x.

## Introduction

Osteoarthritis (OA) is the most common of the musculoskeletal diseases affecting the joints of the hand, knee, hip, and spine [1, 2]. In terms of disability burden, OA is expected to be the fourth leading cause of years lived with disability globally in 2020 [3]. In high-income countries, the medical cost has been estimated to account for between 1% and 2.5% of the gross domestic product [4, 5]. Hand OA (HOA) as a subtype receives relatively little research attention compared with hip and knee OA [6, 7], and yet estimates show a higher prevalence of HOA than other joint sites [8]. The lifetime risk of symptomatic HOA is estimated at 40% with a gender-specific prevalence (1 in 2 women compared to 1 in 4 men) [8, 9]. However, epidemiologic studies of the prevalence of HOA offer wide ranging estimates due to differences in disease definitions, types of populations, and/or risk factors such as genetic factors or environmental exposures across cohorts [6, 10]. When symptomatic, HOA may lead to considerable pain and substantially impacts hand function. Reduced hand function leads to activity limitations and participation restrictions [11, 12]. The etiology of HOA is multifactorial with evidence for the involvement of abnormal mechanical loading and hereditary factors [13]. Comorbidities play a role in the disease burden [14]. There is also a probable contribution of inflammation to pathogenesis [15].

HOA is a heterogeneous disease with involvement of the distal interphalangeal (DIP), proximal interphalangeal (PIP), and joints of the thumb, and more rarely metacarpophalangeal (MCP) joints; however, patients seldom have disease in one anatomical location only [6]. There is a disparity between the radiological and clinical evolution of HOA. Symptomatic disease is considerably less frequent than radiological disease [16], and HOA patients often present varying degrees of symptoms including asymptomatic disease. Presumably, a more severe subset is characterized by radiographic evidence of erosions [13, 16].

Plain radiographs provide the gold standard for morphological assessment of HOA. A posteroanterior radiograph of both hands on a single film/field of view is adequate for diagnosis. Classical features are joint space narrowing (JSN), osteophyte, subchondral bone sclerosis, and subchondral cyst; subchondral erosion occurs in erosive HOA [17]. Diagnosis based on a single radiographic feature (e.g., the presence of JSN or osteophytes) has limited value, whereas the presence of multiple features, especially a composite of clinical and radiographic changes, dramatically improves diagnostic certainty [18].

Previously, we assessed the magnitude and the determinants of the esthetic discomfort generated by HOA using the baseline data of a cohort of 203 patients diagnosed with HOA, the LIège Hand Osteoarthritis Cohort (LIHOC) [19]. These patients were subsequently followed in an observational study over a 2-year period. The objective of the present study is to measure the magnitude and the determinants of clinical and radiological progression in these patients during the prospective follow-up. This study may aid the early identification of HOA patients who may benefit most from early intervention. A greater understanding of the disease time course will support the development of targeted treatments with existing and new therapeutic strategies [6, 20].

## Materials and methods

### Patients

Between February 2013 and August 2014, 203 consecutive outpatients attending a tertiary care center (specialized unit for musculoskeletal health, University Hospital, Liège, Belgium) and diagnosed with HOA according to the American College of Rheumatology x-ray/clinical criteria [21] were included in the LIHOC cohort after giving informed consent. We followed this population for 2 years. Patients were excluded from the study due to refusal or inability to complete the questionnaires. The study design and baseline characteristics of the LIHOC cohort were previously described [19]. This study followed the principles of the Declaration of Helsinki. The local Institutional Review Board approved this trial. At baseline, demographic and clinical characteristics of the population were recorded according to a standardized case report form.

### Clinical assessments

Patient global assessment of hand pain was recorded using a 100-mm visual analog scale (VAS). The Australian/Canadian Osteoarthritis Hand Index (AUSCAN) was used to evaluate disability outcome due to HOA. The AUSCAN is a patient self-reported 15-item questionnaire measuring the severity of three HOA symptoms (pain, stiffness, and function) [22, 23]. The three dimensions were assessed using the VAS version (normalized total score range 0–300, lower values reflect a better health status) [22]. The Osteoarthritis Research Society International (OARSI) recommends the use of the AUSCAN scale in clinical studies conducted in patients with HOA [24].

A single physician (A.N.) performed the clinical and radiographic assessment at the inclusion visit, and at 1 year and 2 years from this time zero. Clinical joint examination was performed by an experienced and purposely trained specialist physician who recorded the number of painful (at rest or upon pressure) and swollen joints. This count included all DIP and PIP and the first MCP joint (thumb base). Clinical presence of Heberden’s and Bouchard’s nodes was recorded.

### Radiographic assessments

Antero-posterior X-rays were obtained, for both hands, at baseline and after 2 years of follow-up. Patients were given a frontal X-ray, and both hands were scanned simultaneously. X-rays were scored using the Kellgren-Lawrence (KL) grading system from grade 0 (none) to 4 (severe). The following joints were included: DIP (4 × 2), PIP (5 × 2), MCP (5 × 2), and joints of the thumb (2 × 2); total number of joints included = 32. Both hands were counted together to obtain an overall score between 0 and 128 [25]. We also applied the Verbruggen-Veys (VV) anatomical scoring system (range: 0–218.4) [26]. For the latter, five anatomical phases are defined: i.e., N = normal, S = stationary joint, J = partial or complete loss of joint space, E = erosive stage, and R = remodeled stage. We also assessed the presence of erosive OA, defined by VV as at least one joint in E or R phase. The intrareader test–retest reproducibility of the X-ray reading was tested on 32 randomly selected pairs of radiographs. The intraclass correlation coefficient ranged between 0.72 and 0.99 for both the KL and the VV scores, indicating good to very good reliability [19, 27]. Films were presented paired to a single reader with known chronology but blinded for patient characteristics [28].

### Clinical and radiographic progression

We defined clinical progression as deterioration in the total AUSCAN score greater than or equal to the minimal clinically important difference (MCID), which was defined as a change of 15 units by Bellamy et al. in 2015 [29]. We also evaluated the proportion of patients with clinically relevant changes in pain and functional limitations, based on the MCID values for the AUSCAN pain and function subscales; these values correspond to 8 and 4 units, for pain and function, respectively [29]. Patients with changes in the AUSCAN pain and function above these values were classified as deteriorated.

We used three separate and independent approaches to define a radiographic progression:

- The detection of at least one new erosive or remodeled joint (phase E or R), on the VV scale, in accordance with the suggestion of Courties et al. [30];
- The observation in at least one joint, of a progression of at least one anatomical stage, using the VV score, and excluding the progression from an N to S stage, as previously described by Meersseman et al. [31];
- A change in the KL total score above the score considered as the smallest detectable difference ([SDD] = 3.215) for this scale [32].

Radiological and clinical parameters recorded at baseline as potential determinants of a clinical and/or radiological progression of HOA included: age, sex, menopause, BMI, waist circumference, self-reported comorbidities (including diabetes, hypertension, dyslipidemia, coronary heart disease, depression and stroke), alcohol consumption, tobacco use, family history of HOA, other location of OA, number of the erosive joints, VV and KL hand scores, number of the swollen hand joints, presence and number of Heberden’s and Bouchard’s nodes, number of the painful hand joints at rest and under pressure, AUSCAN score. At 2 years, the observer was blinded for scores calculated at baseline except for the radiographic analysis.

### Statistical analysis

The data were analyzed using Statistica, version 13, on a Windows platform. The level of statistical significance was set at *p* <  0.05. Demographic and clinical characteristics were analyzed by using descriptive statistics. For normality and homogeneity of data, the tests of Shapiro-Wilk and Levene were used and then justified nonparametric statistical analysis.

Continuous variables are described as median with interquartile ranges ([IQR]: P25-P75). In the second table, the radiological variables are also expressed as the means with standard deviation (SD) for the visual relevance. Categorical variables are presented as number and percentage. For the comparison between matched groups, in the case of continuous data, Wilcoxon test was performed.

Logistic regression analyses were performed to determine whether patient characteristics influenced the main outcomes at 2 years (clinical and radiological progression). The optimal cut-off point, allowing to separate the patients who presented a clinically relevant clinical or radiological progression from those who did not, was obtained where the Youden index reached the maximum value (through ROC analysis). A significance level of 0.25 was used to include variables in the regression model before a stepwise selection procedure. *P* value < 0.05 was considered as statistically significant. The odds ratios (OR) associated with each of the identified predictors were determined using logit regression.

## Results

### Study population

Demographic, clinical, and radiological characteristics of the population are summarized in Table 1. Most patients included in the study completed the questionnaire at 2 years (86.7%; *n* = 176). The reasons for premature withdrawal among 13.3% of patients (*n* = 27) were a lack of interest to complete the study (*n* = 20), personal issues (*n* = 5), or death (*n* = 2). The patients who discontinued the study had similar baseline characteristics compared to those who completed the 2-year follow-up (Additional file 1 Table S1).

**Table 1:** Baseline characteristics of patients with hand osteoarthritis (*N* = 203)

Variable	Median (P25-P75)	*n* (%)
Age, years	69 (61 -, 75)	
Female		183 (90.15)
Menopause		177 (88.06)
BMI (kg/m^2^)	25.71 (22.86-, 29.91)	
Obesity (BMI ≥ 30)		36 (17.73)
Waist circumference (cm)	97.5 (85-, 105)	
Duration since onset of HOA symptoms		
≤ 5 years		96 (47.29)
> 5 years		107 (52.71)
Family history of HOA		95 (46.80)
OA diagnosed at other joints		178 (87.68)
Number of the painful hand joint(s)	1 (0-, 4)	
Number of soft tissue swellings	2 (1-, 4)	
Number of Heberden’s and Bouchard’s nodes	9 (5-, 12)	
Number of the tender joints upon pressure	5 (2-, 10)	
Hand pain (VAS 0–100)	50 (29-, 59)	
AUSCAN		
Overall (0–300)	128.99 (71.08-, 182.54)	
Pain (0–100)	45.22 (19.71-, 64.93)	
Stiffness (0–100)	34.78 (7.25-, 69.57)	
Function (0–100)	48.47 (24.80-, 66.67)	
Erosive HOA		83 (40.89)
Number of the erosive joints (phase E or R in VV scale [0–28])	4 (1-, 7)	
VV score (0–218.4)	31.18 (20.86-, 47.97)	
KL score (0–128)	54 (40-, 66)	

*Abbreviations*: *AUSCAN* AUStralian-CANadian Hand Osteoarthritis Index, *BMI* body mass index, *HOA* hand osteoarthritis, *IQR* interquartile range, *KL* Kellgren-Lawrence, *OA* osteoarthritis, *VV* Verbruggen-Veys

### Clinical evolution

In the study population, the number of PIP and DIP nodes was marginally but significantly higher after 2 years (median 10; IQR 7–13) compared with baseline (median 9; IQR 5–12; *p* <  0.01). The other clinical parameters (number of the painful joints at rest or at pressure, number of the swollen joints, and pain VAS) remained unchanged after 2 years. At the level of the entire cohort, we did not observe any statistically significant change after 2 years in the total AUSCAN score or in the pain, function, and stiffness subscales (*p* = 0.18 to 0.39) (Table 2). However, at 2 years, 61 patients (34.7%) reported a MCID worsening of > 15 on the AUSCAN total scale. For the AUSCAN pain and function subscales, we identified 44 patients (25.1%) and 73 patients (41.7%) for whom the reported worsening was greater than the respective MCID values of 8 (pain) and 4 (function) published for these subscales [29].

**Table 2:** Clinical evolution of hand osteoarthritis over 2 years

Variable	Baseline, *n* = 203					At 2 years, *n* = 176					*p*
	Mean	SD	Median	P25	P75	Mean	SD	Median	P25	P75	
Number of the painful hand joint(s)	3.57	6.18	1	0	4	3.34	5.73	1	0	4	0.83
Number of soft tissue swellings	3.29	3.23	2	1	4	3.10	4.18	2	0	4	0.28
Number of Heberden’s and Bouchard’s nodes	8.80	5.23	9	5	12	10.06	4.96	10	7	13	0.00
Number of the tender joints upon pressure	7.86	8.60	5	2	10	7.82	8.64	4	2	11	0.53
Hand pain (VAS 0–100)	44.15	23.70	50	29	59	42.94	27.67	50	20	65	0.85
AUSCAN											
Overall (0–300)	130.07	77.25	128.99	71.08	182.54	126.80	78.18	127.92	59.36	195.02	0.39
Pain (0–100)	44.27	27.35	45.22	19.71	64.93	41.48	27.27	41.45	16.23	65.80	0.18
Stiffness (0–100)	39.90	33.10	34.78	7.25	69.57	37.35	31.08	33.33	7.25	68.12	0.24
Function (0–100)	45.90	27.02	48.47	24.80	66.67	47.97	28.15	48.95	24.80	73.27	0.18

*Abbreviations: AUSCAN* AUStralian-CANadian Hand Osteoarthritis Index, *SD* standard deviation, *VAS* visual analog scale

Applying a stepwise logistic regression analysis including all potential determinants listed in the “Material and Methods” section, the only statistically significant predictor of worsening after 2 years of the total AUSCAN above its MCID value was a baseline value of the AUSCAN pain subscale < 74.5 (odds ratio [OR] 1.02 [1.01, 1.03]; *p* <  0.01). Similarly, a clinically meaningful worsening of the AUSCAN pain subscale was observed in patients presenting with a baseline value on the AUSCAN pain subscale < 47.0 (OR 1.03 [1.01, 1.04]; *p* < 0.01). The presence of at least four erosive joints at baseline (OR 2.26 [1.07, 4.78]; *p* = 0.03) and a value < 56.0 for the AUSCAN function subscale at baseline (OR 1.02 [1.01, 1.03]; *p* < 0.01) were identified as significant predictors of an increase in the AUSCAN function subscale greater than the MCID after 2 years.

### Radiological evolution

In the whole population, all radiographic scores (i.e., VV score, KL score, the percentage of patients with erosive HOA, and the number of the erosive joints per patient) deteriorated significantly over 2 years (*p* < 0.05) (Table 3).

**Table 3:** Radiological evolution of hand osteoarthritis over 2 years

Variables	Baseline, *n* = 203					At 2 years, *n* = 176					*p*
	Mean	SD	Median	P25	P75	Mean	SD	Median	P25	P75	
VV score (0–218.4)	37.61	27.01	31.18	20.86	47.97	46.40	29.51	37.45	26.66	59.62	< 0.01
KL score (0–128)	52.49	20.17	54	40	66	60.04	19.67	59	49	74	< 0.01
Number of the erosive joints (phase E or R, VV score)	1.98	3.48	0	0	2	2.36	3.94	0	0	4	< 0.01
Erosive HOA	40.91%					44.32%					< 0.01

*Abbreviations: KL* Kellgren-Lawrence, *SD* standard deviation, *VV* Verbruggen-Veys

At the individual level, we identified 39 patients (22.2%) with at least one new erosive joint, 98 patients (57%) with at least one joint progressing by at least one anatomical phase using the VV grading system and 133 patients (75.6%) with a change in KL total score above the SDD (> 3.215) [32]. After a stepwise logistic regression, we identified the presence of at least 4 swollen joints at baseline (OR 2.78 [1.21, 6.39]; *p* = 0.02) and the diagnosis of an erosive OA at baseline (OR 13.23 [5.07, 34.56]; *p* < 0.01) as significant predictors of the propensity to develop at least one new erosive joint during the 2 years of the follow-up. The risk of seeing at least one joint with a progression of at least one anatomical phase on the VV scale during the follow-up was increased in patients aged ≥ 70 years at baseline (OR 1.07 [1.02, 1.12]; *p* < 0.01) and by the diagnosis of erosive OA at baseline (OR 10.60 [4.63–24.25]; *p* < 0.01). A change in the KL total score above 3.215, corresponding to the SDD for this grading scale [32] was statistically more frequent in patients who had at least one painful joint at baseline (OR 3.43 [1.68, 7.01]; *p* < 0.01).

## Discussion

In this study, we followed a cohort of patients with HOA for 2 years to measure the extent and rate of clinical and radiological progression, and we sought to identify predictors of clinical and radiological worsening of HOA during this period. In the whole population, we observed a statistically significant progression in all the radiographic indices of severity, while only one clinical parameter (number of nodes) worsened over time (from an average of 9 to 10 nodes). At the individual level, the number of patients whose progression was above the thresholds previously reported as clinically meaningful ranged from 25 to 42% (clinical progression) and from 22 to 76% (radiological progression). A low baseline value on the AUSCAN pain subscale was identified as the only significant predictor of meaningful clinical worsening of HOA over 2 years (*p* < 0.01). The presence of erosions and the swollen joints at baseline were found to be predictive of clinically meaningful radiological progression of HOA over 2 years.

Our finding of a difference in the time course of the clinical and radiological features of OA is not surprising and has been reported previously in patients with HOA [33, 34] and knee OA [35]. Among patients with HOA followed for 2 years, radiological progression was not associated with changes in self-reported pain and function [36]. In longer studies following patients with HOA for 6 years [31, 34] or 7 years [33], radiological progression was associated with different clinical outcomes including a small increase in pain or functional limitations [34], symptomatic steady-state [31], or even a decrease in tender joints [33].

In subjects whose AUSCAN (total and pain) score changes were above the threshold considered as clinically relevant, we identified a lower level of baseline pain (a value of the AUSCAN pain subscale below 74.5) as the only significant predictor of clinical worsening. Patients with less pain and less functional impact at baseline were more likely to be significantly affected 2 years later. The absence of worsening during follow-up in patients presenting a high level of pain at baseline (> 74.5 on a 0–100 VAS) may reflect a ceiling effect. In a previous study by Bijsterbosch et al. [34], 50% of patients had clinical progression after 6 years, a figure which compares well with our finding that 25% of patients had a clinically relevant increase in pain after 2 years. Poor outcome for pain in the study by Bijsterbosch et al. was related to high level of functional limitations and a higher number of painful joints at baseline [34]. The younger age (average 10 years younger) and the lower level of pain at baseline compared with our population, as well as the use of a patient-reported outcomes measure (Patient Acceptable Symptom State) to assess symptomatic progression may be factors that explain the divergence with our results.

We report that the presence of the erosive joints at baseline was significantly associated with poor evolution of the AUSCAN functional score after 2 years; this is in accordance with recently published results [37]. In a follow-up of 112 patients with HOA over 7 years, Haugen et al. showed associations between radiographic features and measures of pain and physical function in HOA. In their study, incident rather than prevalent erosive OA was associated with clinical worsening [33]. These findings were confirmed in a study assessing pain and radiological progression over 5 years, in patients with erosive and non-erosive HOA [38]. In comparison, our patients were, on average, 8 years older and presented with a greater number of erosive joints than those described in their article [33]. In our study, the number of tender joints at baseline did not emerge as a significant predictor of clinical progression, as was the case during the 7-year follow-up study [33]. Conversely, the radiological evolution of our patients was negatively influenced by the presence, at baseline, of a high number of swollen joints, by the presence of painful joints, and by the presence of an erosive OA. It should also be noted that older patients (> 70 years) had a greater propensity for VV progression during follow-up.

Our results are supported by several previous publications. The value of the presence of erosive and tender joints as predictors of a significant radiological progression of HOA was already reported [35, 36, 39]. The rather constant description of an impaired prognosis in patients presenting with erosive OA even generated a debate on whether erosive HOA should be considered as a severe form of radiographic HOA or as a distinct phenotype of the disease [37, 39, 40]. Haugen et al. concluded that erosive OA is characterized by more severe radiographic progression compared with non-erosive OA [38]. Similarly, the presence of erosive OA was associated with radiographic progression during a 6-year follow-up [34], and painful joints were linked to a more severe radiographic outcome, a feature that we also report for the progression of the KL score. We did not, as reported in their study, find a predictive role for baseline nodes and radiological progression although the duration of our follow-up was shorter. Erosive OA has also been associated with a higher number of nodes and a worse outcome in terms of pain and functional limitations [41].

The presence of at least 4 swollen joints at baseline in our study was significantly associated with the development of erosive OA within 2 years. Soft tissue swelling was the only clinical variable associated with erosive radiographic progression over 5.8 years in a similar study. The 2.56 increased risk reported for this association compares well with the OR of 2.78 for erosive joint emergence in patients with swollen joints at baseline in our study [31]. We also observed that older patients (≥ 70 years) had a modest increased risk of radiographic progression compared to younger individuals, a population that is not frequently included in studies of HOA progression [31, 33, 34, 38]. In a report combining cross-sectional comparisons of age-specific cohorts of 70, 75, and 79 years old and a longitudinal study including individuals aged 75–79 years, a modest but non-significant progression of HOA was observed after 4 years as assessed by KL score, which suggested reduced HOA progression after 75 years of age compared with the earlier age groups [42].

Whereas previous reports have emphasized the role of comorbidities as risk factors for HOA incidence and/or poor outcomes [14, 30, 39, 43], we did not find significant associations between individual comorbidities or the total number of comorbidities recorded at baseline and HOA progression.

The strengths of the present study include the recruitment of a sample of patients, which, even if rather limited to identify mild correlations, compares well with previously published studies. The diagnosis of HOA followed a widely accepted definition [21]. The proportion of patients who completed the 2-year follow-up was high at 86.7%. We were able to confirm that the patients who prematurely discontinued the study were not different from those who completed the last questionnaire, thus excluding a reporting bias. We included many variables in our search for progression predictors. We based our analyses on cut-off values considered to reflect relevant disease progression both for the AUSCAN [29] and for radiographic assessments [30–32]. As recommended, one single reader scored all radiographs at baseline and after 2 years and the serial images were read with the images presented with known chronology [44].

The limitations of our investigation include a putative selection bias. The recruitment of patients from one tertiary care center (University Hospital of Liège), which may invalidate extrapolation of our results to the entire HOA population. Comorbidities were included based on self-reported diseases and were not confirmed by medical records or drug prescriptions. The limited size of our sample and the relatively short duration of our follow-up might also explain the absence of correlation between comorbidities and HOA progression. It should also be noted that our population presented a higher proportion of erosive OA compared with previous samples [45]. As previously mentioned [34], the follow-up assessment took place at 1 and 2 years after the baseline measurement taken at the inclusion visit (time 0) (between 2013 and 2014). The changes observed at the end of study visit may not accurately reflect the evolution of the disease over the whole duration of the follow-up. However, on average, they can be considered as a reliable picture of HOA progression [34].

In future, more information could be gained by separately analyzing the thumb base and interphalangeal joints. The incidence and progression of thumb base OA was previously linked to specific risk factors and this location is associated with high clinical burden [46]. It would also be interesting to compare the evolution of a large sample of patients with non-erosive or erosive HOA [38]. Linking clinical and radiological progression at the individual level, with repeated assessments at shorter intervals and over a longer period would help us to better understand the time course of HOA.

## Conclusion

In conclusion, we observed radiological progression of HOA in our cohort over 2 years, whichever assessment scale was used, and without evidence for clinical worsening of the AUSCAN composite measure of pain, function, and stiffness, suggesting an absence of correlation between the two measures of disease progression. However, at the individual level, we identified a subset of our population who experienced a relevant clinical and/or radiological worsening of HOA. The proportion of progressors varied between 25% and 75% of the entire sample based on the tool selected to define progression. Some baseline features were identified as significant predictors of greater HOA progression including pain level for clinical progression and the presence of erosive or swollen joints for radiological progression. These results support previous publications and may aid the early identification of HOA patients who may benefit most from early intervention.

## Supplementary Information

**Additional file 1: Table S1.** Baseline characteristics of patients who completed the study (*n* = 176) versus those who did not for any reason (*n* = 27).

## Data Availability

The datasets used and analyzed during the current study are available from the corresponding author on reasonable request.

## Abbreviations

AUSCAN: Australian/Canadian Osteoarthritis Hand Index
DIP: Distal interphalangeal
HOA: Hand osteoarthritis
IQR: Interquartile range
KL: Kellgren-Lawrence Grading System
LIHOC: Liège Hand Osteoarthritis Cohort
MCID: Minimal clinically important difference
MCP: Metacarpophalangeal
OA: Osteoarthritis
OARSI: Osteoarthritis Research Society International
OR: Odds ratio
PIP: Proximal interphalangeal
SD: Standard deviation
SDD: Smallest detectable difference
VAS: Visual analog scale
VV: Verbruggen-Veys anatomical scoring system
