# Supplementary material for: The presence of erosive joints is a strong predictor of radiological progression in hand osteoarthritis: results of a 2-year prospective follow-up of the Liège Hand Osteoarthritis Cohort (LIHOC)
Source: Arthritis Res Ther. 2021 Jan 6;23:12. doi: 10.1186/s13075-020-02390-x (PMC7788934; doi:10.1186/s13075-020-02390-x)
Supplement: Supplementary file 1 — Additional file 1: Table S1. Baseline characteristics of patients who completed the study (n = 176) versus those who did not for any reason (n = 27). [file 13075_2020_2390_MOESM1_ESM.docx]

**Additional file 1.**

**Table S1** Baseline characteristics of patients who completed the study (n = 176) versus those who did not for any reason (n = 27)

| Variable | Patients who completed (n = 176) | | Patients who discontinued (n = 27) | | \| *p* \| \| --- \| |
| --- | --- | --- | --- | --- | --- | --- |
|  | \| Mean \| \| --- \| | \| SD \| \| --- \| | \| Mean \| \| --- \| | \| SD \| \| --- \| |  |
| \| Age \| \| --- \| | 67.90 | 9.08 | 70.30 | 9.88 | 0.21 |
| \| BMI \| \| --- \| | 26.15 | 4.59 | 25.59 | 4.97 | 0.56 |
| \| Waist circumference (cm) \| \| --- \| | 96.76 | 15.55 | 94.52 | 14.83 | 0.48 |
| \| Duration since the onset of HOA symptoms \| \| --- \| | 2.81 | 0.95 | 2.48 | 1.05 | 0.10 |
| \| Hand pain (VAS 0-100) \| \| --- \| | 44.15 | 23.70 | 40.74 | 26.93 | 0.49 |
| Number of painful hand joint(s) | 3.57 | 6.18 | 2.07 | 2.96 | 0.22 |
| Number of soft tissue swellings | 3.29 | 3.23 | 2.04 | 1.79 | 0.06 |
| Number of Heberden's and Bouchard's nodes | 8.80 | 5.23 | 10.85 | 6.63 | 0.07 |
| Number of tender joints upon pressure | 7.86 | 8.60 | 5.37 | 6.05 | 0.15 |
| \| AUSCAN total (0-300) \| \| --- \| | 130.07 | 77.25 | 126.80 | 64.89 | 0.83 |
| \| Number of erosive joints \| \| --- \| | 1.98 | 3.48 | 2.00 | 4.16 | 0.98 |
| \| VV score (0-218.4) \| \| --- \| | 37.61 | 27.01 | 39.12 | 27.75 | 0.79 |
| \| KL score (0-128) \| \| --- \| | 52.49 | 20.17 | 54.48 | 18.91 | 0.63 |

*Abbreviations* : *AUSCAN* AUStralian-CANadian Hand Osteoarthritis Index, *BMI* body mass index, *HOA* hand osteoarthritis, *KL* Kellgren-Lawrence, *SD* standard deviation, *VV* Verbruggen-Veys
